# Current therapeutic landscape and resistance mechanisms to larotrectinib

**DOI:** 10.20892/j.issn.2095-3941.2023.0471

**Published:** 2024-02-05

**Authors:** Weiji Xie, Jiaqian Xu, Suying Lu, Yizhuo Zhang

**Affiliations:** 1 Department of Pediatric Oncology, State Key Laboratory of Oncology in South China, Guangdong Provincial Clinical Research Center for Cancer, Collaborative Innovation Center for Cancer Medicine, Sun Yat-sen University Cancer Center, Guangzhou 510060, China; 2 Young Talents Program of Sun Yat-sen University Cancer Center, Guangzhou 510060, China

Cancer has emerged as the leading cause of mortality worldwide and is driven by numerous intricate factors^1^. The tropomyosin receptor kinase (TRK) proto-oncogene family consists of the TRKA, TRKB, and TRKC transmembrane receptors, which are encoded by the *NTRK1, NTRK2*, and *NTRK3* genes, respectively, and are widely expressed in the nervous system and many non-neuronal tissue types^2^. TRK signaling plays crucial roles in neuronal development, differentiation, and diverse neuronal functions, such as neuronal survival, synapse formation and plasticity, and axon and dendrite formation, in physiologic processes^3^. Fusion of the 3’ region of the NTRK gene with the 5’ end of unrelated gene partners through chromosomal translocations represents a major genomic alteration with oncogenic potential^4^. Newly formed cancer proteins are usually constitutively active or overexpressed kinases, promoting continuous signal transduction and contributing to tumorigenesis^4^. *NTRK* gene fusions have been identified in diverse pediatric and adult malignancies^5^, particularly at higher frequencies in rare pediatric tumors, including infantile fibrosarcoma, congenital mesoblastic nephroma, and papillary thyroid cancer.

Larotrectinib (LOXO-101) is an orally administered and highly selective small-molecule inhibitor of all three TRK proteins with potent inhibitory effects *in vitro* and higher selectivity for TRK than for other kinases. Recent phase I/II trials have demonstrated overall favorable and durable responses in patients with TRK fusion-positive solid tumors^6–8^. Although notable achievements in the application of TRK therapies to treat different cancers, resistance will be developed in some cancers with TRK fusion mutations.

## Clinical activity of larotrectinib

The activity of larotrectinib in patients with NTRK fusion-positive tumors has been investigated in three clinical trials.

In an initial phase I study (NCT02122913) involving adults with NTRK fusion-positive tumors, larotrectinib exhibited a remarkable overall response rate (ORR) of up to 88%^7^. A dose of 100 mg BID was recommended for phase II trials. Furthermore, a pharmacokinetic analysis of larotrectinib was conducted in a phase I/II study [SCOUT (NCT02637687)] involving pediatric and adolescent patients between 1 month and 21 years of age^8^. Responses to larotrectinib were observed in infants, children, and adolescents irrespective of the tumor type or NTRK gene fusion with an impressive ORR of 93%^8^. Based on the promising responses to larotrectinib observed in patients with NTRK gene fusions, a tumor-agnostic phase II basket trial [NAVIGATE (NCT02576431)] was initiated for patients with NTRK gene fusions^6^. This comprehensive analysis included 55 patients, spanning a wide age range from 4 months to 76 years and covering 17 distinct TRK fusion-positive tumor types. The ORR was 75% as assessed independently and 80% as evaluated by the investigators. Notably, at the 1-year mark, 71% of the responses were still ongoing and 55% of the patients remained progression-free. Larotrectinib exhibited significant antitumor activity across all age groups and diverse tumor types, irrespective of the specific NTRK gene or fusion type.

Based on the findings of these three clinical trials, a comprehensive evaluation of the efficacy and safety of larotrectinib was conducted by analyzing data from specific patient subgroups of interest. In a subgroup of 24 patients diagnosed with TRK fusion-positive salivary gland cancers, larotrectinib was shown to have sustained efficacy and a favorable safety profile, with an ORR of 92% and a progression-free survival rate of 78% at the 24-month follow-up evaluation^9^. Within an expanded dataset involving pediatric patients with TRK fusion-positive cancers, the majority of children achieved a partial or complete response within 1–2 treatment cycles with larotrectinib^10^. Moreover, larotrectinib reduces the risk of amputation compared to the standard of care in patients with infantile fibrosarcoma^11^. In a subgroup of patients with non-primary central nervous system (CNS) solid tumors and brain metastases, or primary CNS tumors harboring NTRK gene fusions, larotrectinib had confirmed responses and long-lasting disease control, highlighting the antitumor efficacy in patients with intracranial diseases^12^. Data from a cohort of 33 patients with primary CNS tumors indicated relatively lower efficacy compared to non-CNS tumors with an ORR of 30%^13^.

## Resistance to larotrectinib

The mechanism underlying tyrosine kinase inhibitor (TKI) resistance can be categorized into two main groups: on-target mechanisms, which involve mutations in the kinase domain; and off-target mechanisms, which involve activation of parallel bypass pathways^14^. While current phase I/II clinical trials involving larotrectinib in patients with NTRK fusion-positive tumors show promising therapeutic effects, resistance to larotrectinib is thought to develop over time as occurs with other TKIs, thus limiting long-term benefits. A summary of the known resistance mechanism mutations and treatment strategies for larotrectinib resistance is presented in **Table 1**.

**Table 1:** Summary of known resistance mechanisms, mutations, and treatment strategies after resistance to larotrectinib

Resistance mechanism	Known resistance mutations sites		Treatment strategy
On-target resistance	Solvent front	NTRK1^G595R^, NTRK2^G639R^, NTRK2^G639L^, NTRK3^G623R^, NTRK3^G623E^	Next-generation TRK inhibitors
	Gatekeeper residue	NTRK1^F589L^, NTRK2^F633L^, NTRK3^F617L^	
	xDFG motif	NTRK1^G667C^, NTRK1^G667S^, NTRK2^G709C^, NTRK3^G696A^	
Off-target resistance	BRAF mutation	BRAF^V600E^	Targeted combination therapy
	BRAF rearrangement	BRAF-CREB3L2 rearrangement, intergenic/BRAF rearrangement	
	BRAF amplification	BRAF-exon9_exon18 amplification	
	KRAS mutation	KRAS^G12D^, KRAS^Q61H^	
	EGFR amplification	NA	
	IGF1R activation	NA	

## On-target resistance to larotrectinib

Mutations within the NTRK domain confer resistance to TRK inhibitors by causing steric hindrance to inhibitor binding, inducing conformational changes in the kinase domain, or altering the ATP-binding affinity^15^. These kinase domain mutations lead to amino acid substitutions that affect the following three principal regions: solvent front; gatekeeper residue; and xDFG motif. The solvent-front substitutions comprise NTRK1^G595R^, NTRK2^G639R^, and NTRK3^G623R^. The gatekeeper substitutions include NTRK1^F589L^, NTRK2^F633L^, and NTRK3^F617L^. The xDFG substitutions consist of NTRK1^G667C^, NTRK1^G667S^, NTRK2^G709C^, and NTRK3^G696A^.

The first two patients have been reported to have developed acquired resistance to larotrectinib^16^. After an initial positive response to larotrectinib, a 55-year-old woman with advanced LMNA-NTRK1 fusion-positive colorectal cancer and a 2-year-old girl with ETV6-NTRK3 fusion-positive recurrent infantile fibrosarcoma had disease progression. Subsequent molecular testing revealed the presence of a solvent front (TRK1^G595R^) mutation in the patient with colorectal cancer and a solvent front (TRK3^G623R^) mutation in the patient with infantile fibrosarcoma. To precisely delineate the frequency of major resistance mechanisms, a retrospective analysis was performed involving 18 patients with 9 distinct fusions and 10 different tumor types^17^. Among patients with NTRK fusion-positive cancer, on-target resistance (15/18) preferentially involved the solvent front (13/15) and occurred more frequently than off-target resistance (2/18) to first-generation TKI therapy.

Detailed molecular detection of drug resistance and X-ray crystallography has facilitated the development of next-generation TKIs to overcome on-target resistance to earlier-generation TKIs. LOXO-195 (selitrectinib), a second-generation TRK inhibitor, was designed to be a clinical candidate for prevention of the acquired resistance to larotrectinib observed in clinical trials. By conducting targeted mutagenesis experiments and drawing insight from structurally analogous oncogenic kinases, such as anaplastic lymphoma kinase (ALK) and c-ros oncogene 1 (ROS1), researchers have predicted the structural basis for acquired resistance to larotrectinib exposure. In fact, preclinical validation of the efficacy of LOXO-195 against resistance to first-generation NTRK inhibitors was achieved ahead of clinical data.

Clinical phase I trials of LOXO-195 (NCT03215511) and expanded access experience (NCT03206931) have been explored^18^. A total of 31 patients (7 children and 24 adults) with 11 identified TRK fusion cancer types and resistance to prior TRK inhibitor therapy received LOXO-195. In a patient cohort with kinase domain mutations [*n* = 20 (solvent front, *n* = 14; gatekeeper, *n* = 4; and xDFGs mutations, *n* = 2)], the ORR was 45% (9/20). Notably, among the three patients with resistance caused by off-target bypass mutations, no positive response to LOXO-195 was observed; two patients had progressive disease and one patient was non-evaluable. Moreover, a corollary study indicated that the sequential utilization of second-generation therapy may modify the evolutionary kinetics of mutation retention and acquisition^17^. In two patients exhibiting solvent-front mutations and progression following second-generation TRK inhibition, one patient with an initial NTRK1^G595R^ mutation developed resistance through acquisition of KRAS^G12A^ and NTRK1^G667A^ alterations, as well as NTRK1^G595R^ loss. Another patient harboring a NTRK3^G623R^ mutation developed an NTRK3^F617I^ gatekeeper mutation with NTRK3^G623R^ loss.

In summary, on-target resistance mutations lead to amino acid substitutions involving the solvent front, gatekeeper residue, and xDFG motif, which sterically interfere with first-generation TRK inhibitor binding. Therefore, the frequency of on-target resistance in TRK fusion-positive cancers should be determined in a larger number of patients. Next-generation TRK inhibitors have been designed to address on-target resistance mutations while maintaining potency against all three TRK kinases. These clinical findings emphasize and encourage repeated longitudinal molecular profiling for the management of patients with cancer.

## Off-target resistance to larotrectinib

Like other activated oncogenic tyrosine kinases, TRK fusion cancers can undergo genomic alterations involving other receptor tyrosine kinases or downstream pathway mediators, which are referred to as off-target mechanisms. Patients resistant to such alterations often do not respond to second-generation TRK inhibitors because the mutations do not involve the kinase domain. Currently, there is no consensus regarding the frequency of off-target resistance in patients receiving larotrectinib therapy. Only three studies have investigated the mechanisms underlying larotrectinib off-target resistance.

The first study investigating the mechanisms underlying larotrectinib off-target resistance was prospective and conducted by Cocco et al.^19^. Within the framework of prospective clinical trials, the authors systematically collected paired sequencing data from tumor biopsies and circulating cell-free DNA to identify patients with drug resistance that was not attributable to TRK kinase domain mutations. This study provided a comprehensive overview of clinical progression in patients with off-target resistance, analyzed the molecular profiling results, performed a series of cytological experiments for validation, and demonstrated that activation of the mitogen-activated protein kinase (MAPK) pathway represents a recurrent and convergent mechanism underlying off-target bypass resistance. Herein we present two patients to illustrate the validation process conducted by the research team.

The first patient was diagnosed with CTRC-NTRK1 fusion-positive pancreatic cancer and developed off-target resistance after 5 months of larotrectinib therapy, acquiring BRAF^V600E^ and KRAS^G12D^ mutations through paired sequencing. Similar resistance, along with the emergence of a BRAF^V600E^ mutation, has been observed in patient-derived xenografts (PDXs) and cell lines following larotrectinib administration. Subsequently, the patient was switched to a second-generation TRK inhibitor; however, the condition progressed rapidly. A cell viability assay showed that the ectopic expression of BRAF^V600E^ in an NTRK1 fusion-positive pancreatic cancer cell line maintained a strong proliferation trend under LOXO-195 treatment conditions. Upon detection of the emergent BRAF^V600E^ mutation, triple therapy (a combination of RAF and MEK inhibitors, and larotrectinib) was administered. This treatment regimen led to rapid tumor regression accompanied by a reduction in the allele frequencies of NTRK fusion and BRAF^V600E^. Similar inhibition was observed in PDXs. Furthermore, the research team found that administering upfront combination therapy resulted in more durable tumor regression than sequential TRK inhibitor monotherapy.

The second case was diagnosed as an LMNA-NTRK1 fusion-positive colorectal cancer, which developed on-target resistance *via* acquisition of an NTRK1^G595R^ solvent front mutation. Administration of LOXO-195 restored disease control, but subsequently resulted in liver progression with a KRAS^G12A^ substitution in the first liver metastatic lesion and a new KRAS^G12D^ substitution in the second relapse after ablation of the liver metastasis and LOXO-195 continuation. Extended treatment of an LMNA-NTRK1-positive NTRK1^G595R^-mutant colorectal cancer cell line with LOXO-195 confirmed the acquisition of KRAS^G12D^. Furthermore, the ectopic expression of KRAS^G12A^ and KRAS^G12D^ in colorectal cancer cell lines was sufficient to activate the MAPK pathway and maintain high cell viability in the presence of LOXO-195.

Lu et al. were pioneers in reporting a series of off-target resistance cases to larotrectinib among pediatric patients^20^. By conducting prospective paired sequencing, the research team concluded that the off-target resistance mechanism underlying larotrectinib in pediatric patients is associated with activation of the MAPK pathway, which is consistent with findings in adult patients. Herein we describe two rare cases of off-target larotrectinib resistance in pediatric patients.

The first patient, a 5-year-old with TP53-NTRK1 fusion-positive infant-type hemispheric glioma, initially exhibited a positive response to larotrectinib, but had a relapse after 10 treatment cycles. Genomic profiling revealed EGFR amplification and a CDKN2A/B homozygous deletion. The second patient, an 11-year-old with an LMNA/NTRK1 rearrangement low-grade malignant spindle cell tumor, also had disease progression following standard therapy. Larotrectinib initially showed a satisfactory effect on tumor progression after 20 cycles of treatment. Repeat biopsies revealed new genomic alterations, including a BRAF-CREB3L2 rearrangement, intergenic/BRAF rearrangement, BRAF-exon9_exon18 amplification, CDKN2A/B homozygous deletion, MTAP deletion, and RPTOR amplification. These newly emerged genomic alterations strongly activated the RAF-MEK-ERK signaling pathway and mediated the acquisition of resistance. Furthermore, the research team hypothesized that the CDKN2A/B homozygous deletion detected in both cases potentially contributed to the occurrence of off-target resistance to larotrectinib. Further research is necessary to validate this finding.

Moreover, Fuse et al. identified the potential mechanisms underlying resistance to TRK inhibitors and explored potential therapeutic strategies to overcome this resistance^21^. TRK inhibitor-resistant cells were established and drug screening was performed using multiple TKIs. Insulin-like growth factor receptor type 1 (IGF1R)-mediated resistance to TRK inhibitors was detected with the establishment and analysis of resistant KM12 cells. Combination therapy targeting IGF1R and NTRK was shown to effectively reverse this resistance.

Overall, existing evidence indicates that mutations in bypass pathways are associated with off-target resistance to larotrectinib, including activation of the MAPK pathway in adults and children, and IGF1R activation. Further research is warranted to confirm the mechanism underlying the activation of off-target resistance to larotrectinib *via* other kinases or bypass pathways. Next-generation TRK inhibitors cannot adequately overcome the resistance mediated by off-target mechanisms because next-generation TRK inhibitors do not involve mutations in the kinase domain. However, targeted combination therapies have demonstrated the potential to reestablish tumor control. Exploring different drug combinations and administration sequences provides additional possibilities for clinical medication treatment.

## Conclusions

Major advances in sequencing technologies have transformed the paradigm of cancer treatment towards precise oncology. NTRK fusions drive a wide variety of adult and pediatric cancers. While larotrectinib has shown favorable responses in most patients with TRK fusion-positive cancers, acquired resistance limits the long-term benefits. On-target resistance involving the solvent front is more common than off-target resistance to larotrectinib in NTRK fusion-positive cancers. Specifically, off-target mechanism-driven resistance can only be overcome by identifying bypass genetic mutations and administering targeted combination therapies. This highlights the crucial role of molecular sequencing in the management of patients with tumors in precision oncology.
